# Study on the Compressive and Flexural Properties of Coconut Fiber Magnesium Phosphate Cement Curing at Different Low Temperatures

**DOI:** 10.3390/ma17020444

**Published:** 2024-01-17

**Authors:** Zhiwei Lin, Liwen Zhang, Wenzhi Zheng, Xiangyun Huang, Junping Zhang

**Affiliations:** 1 Department of Civil Engineering, Guangzhou University, Guangzhou 510006, China; linzhiwei9801@163.com (Z.L.); wzzheng@gzhu.edu.cn (W.Z.); 2 Digital Intelligence Research Center for Roads and Bridges, Guangzhou University, Guangzhou 510006, China; 3 Earthquake Engineering Research & Test Center, Guangzhou University, Guangzhou 510006, China; eertchxy@gzhu.edu.cn

**Keywords:** coconut fiber, magnesium phosphate cement, static compression test, three-point bending test, curing temperature

## Abstract

The incorporation of coconut fiber (CF) into magnesium phosphate cement (MPC) can effectively improve upon its high brittleness and ease of cracking. In practical engineering, coconut fiber-reinforced magnesium phosphate cement (CF-MPC) will likely work in cold environments. Therefore, it is essential to understand the effects of various types of low-temperature curing on CF-MPC performances, but there are very few studies in this area. In this study, the static compression and three-point bending test were utilized to examine the compressive and flexural characteristics of CF-MPC with various CF contents and different negative curing temperatures. Scanning electron microscopy (SEM) and X-ray diffraction (XRD) were conducted to observe the impact of low-temperature maintenance on the structure and hydration reaction of the specimens. The results indicate that CF-MPC curing at low temperatures was more prone to cracks during compression and bending, while the appropriate amount of CF could enhance its plastic deformation capability. The CF-MPC’s compressive and flexural strength declined as the curing temperature dropped. Moreover, with the rise in CF content, the samples’ compressive strength also tended to fall, and there was a critical point for the change in flexural strength. In addition, MPC’s primary hydration product (MgKPO_4_·6H_2_O) decreased with a drop in curing temperature, and more holes and fractures appeared in CF-MPC.

## 1. Introduction

A novel type of high-performance cementitious material, magnesium phosphate cement (MPC), is extensively employed in building, military, and ecological rapid repair [1,2,3], which is mainly prepared using acid phosphate, ultra-high temperature calcined magnesium oxide with the retarder borax, and other dopants with acid-base neutralization and physical action. MPC possesses the properties of ceramics and the performance of cement simultaneously. It can solidify and harden rapidly and offers the exceptional benefits of frost resistance, strong bonding ability, and high early strength [4,5,6,7,8]. Like typical cementitious materials, MPC has poor fracture toughness, high brittleness, and low tensile strength. These drawbacks make it challenging to employ MPC regularly for structural repair projects. The right amount of artificial fibers, such as polypropylene, basalt, or other fibers, was found to be mixed into MPC to modify it into a fiber-reinforced cementitious composite, which can delay the extension of matrix cracks by transferring the stress effect between the fibers and the MPC. At the same time, the fibers help the matrix withstand some loads [9], causing the MPC to absorb more energy, which improves MPC toughness and crack resistance [10,11,12,13]. The ability of MPC to absorb energy is enhanced, thus improving its toughness. However, the production process of artificial fibers cannot avoid the high cost, high energy consumption, and air-polluting emissions, which is not conducive to human safety and environmental protection as part of sustainable development. In this regard, green and eco-friendly plant fiber is required to effectively enhance MPC performance and eliminate the need for synthetic fibers.

Research investigations have found that coconut fiber (CF) has better tensile strength, toughness, and tensile properties at break compared to other plant fibers [14,15,16], is renewable, green, and low-cost, and has several applications in the direction of fiber-reinforced cementitious composites. When CF was doped into the cementitious matrix by Flávia et al. [17] and Riza et al. [18], the test revealed that the cement matrix’s toughness and flexural capacity rose. Previous studies have found [19,20] that adding the right proportions of CF to MPC may effectively address the material’s high brittleness and poor toughness, as well as increase MPC’s deformation capacity and flexural strength. Due to the positive impact of CF on MPC’s performances, the use of CF-MPC is promising and has high benefits for engineering applications.

In order to ensure the regular servicing of infrastructure construction, it is essential to repair damaged pavements, bridge structures, etc. [21,22]. The actual project may occur in winter, when the outdoor temperature is below 0 °C, and the repair material’s mechanical strength and working performance in cold conditions must be high [23,24]. As a repair material of roads and bridges and other infrastructure construction, CF-MPC has mechanical properties in low-temperature curing that are of great engineering significance, but the current relevant research is sparse. Jie Yuan et al. [25] and Huang et al. [26] studied the mechanical performances of MPC by simulating a cold environment and found that MPC could still hydrate at low temperatures. However, XRD showed that the unhydrated phosphate in MPC increased, and the hydration was inhibited, thus affecting the matrix strength. Jia et al. [27] and Hipedinger et al. [28] explored MPC’s mechanical characteristics and setup time under different negative temperatures, and found that with the reduction of the curing temperature, the MPC mixing water gradually freezes. As the temperature dropped, MPC’s setting time slowed down and internal free water crystallization developed, affecting the material’s hydration process and resulting in a loss of mechanical strength. Hu Feng et al. [29,30] discovered that low-temperature curing degraded the properties of MPC compared to room temperature, but the moderate addition of polyvinyl acetate (PVA) fibers or micro-steel fibers (MSF) improved its plastic deformability and flexural strength. While most of the research above focused on MPC or artificial fiber composites curing in low-temperature settings, few studies examined MPC and natural fiber composites.

With the purpose of providing guidance for the appropriate use of CF-MPC in practical projects, we conducted experimental research on CF-MPC with curing at different low-temperatures and with different CF contents through static compression and three-point bending tests, and obtained the failure modes, test curves, and mechanical strengths. Furthermore, scanning electron microscopy (SEM) and X-ray diffraction (XRD) were both employed to explore the impact of low-temperature curing on the microstructure, hydration products, and CF-MPC’s level of hydration.

## 2. Experimental Programs

### 2.1. Raw Materials and Specimens

The principal raw materials of CF-MPC were reburned magnesium oxide (MgO), fly ash (FA), potassium dihydrogen phosphate (KH_2_PO_4_), borax retarder, and coconut fiber (CF). The potassium dihydrogen phosphate and borax used in the tests were industrial-grade products with a mass ratio of more than 99.95%. The heavy burnt magnesium oxide was provided by Hans Technology Material Co., Ltd. (Yingkou, China), with a fineness of 300 mesh, a specific surface area of 2275 cm^2^/g, and a density of 2.65 g/cm^3^. The fly ash was produced by Gongyi Bairun Refractories Co., Ltd. (Gongyi, China) with a specific surface area of 390 m^2^/kg, fineness of 300 mesh, and density of 2.7 g/cm^3^, as shown in Table 1. The coconut fiber was produced in Sri Lanka, with an average diameter of 250 µm, a tensile strength of 142 ± 15 MPa, and a density of 1.20 g/cm^3^. In the tests, the mixing water was placed in a 0 °C ± 2 °C low-temperature test chamber.

The MPC mixing ratios prepared in this paper’s experiments were based on previous studies [20]. Table 2 shows the mixing ratio parameters of MPC, where the water content was kept constant in MPC specimens, not varying with the CF content. Prior to making MPC specimens, KH_2_PO_4_ and borax need to be dried, ground, and sieved to 42 mesh. CF needs to be cleaned, dried, and cut into 20 mm pieces.

**Figure 1:**
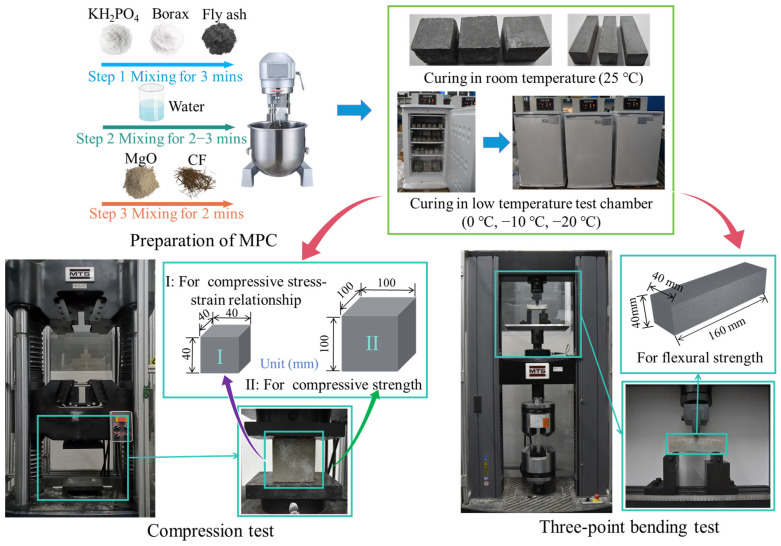
The specimens and the test setups.

As shown in Figure 1, three sizes of specimens were used for the tests in this paper, of which 40 mm × 40 mm × 40 mm was used for testing the compressive strength, 100 mm × 100 mm × 100 mm was used for testing the stress–strain curve, and 40 mm × 40 mm × 160 mm was used for testing the flexural strength. The length of the CF selected for the three trials was 20 mm, and the CF volume content was 0%, 1%, 2%, 3%, and 4%, where fiber content refers to the percentage of the total volume of the sample. The samples were maintained at 25 °C and in low-temperature test chambers (0 °C, −10 °C, −20 °C) for 28 days. For each group of fiber content, three specimens with the same parameters were set up and numbered, in which SS represents the stress–strain curve test, CS represents the compressive strength test, TB represents the flexural strength test, T0 represents the curing temperature of 0 °C, and CF1 represents the 1% fiber content.

### 2.2. Test Setup

Figure 1 shows the details of the test setups. A 100-ton universal testing machine was employed for the stress–strain curve test of the CF-MPC, with a 2.0 mm/min loading rate and a damage sensitivity of 90%. Test data were gathered from the specimen surface strain gauges and were arranged by the testing machine for feedback. The CF-MPC compressive strength test was carried out following the GB/T17671-1999 [31] specification, with a loading displacement rate of 2.0 mm/min, setting the 60% peak load as the failure sensitivity. The three-point bending test was conducted using a 30-ton universal testing machine with a displacement loading speed of 1.0 mm/min and a failure sensitivity of 10%. The mechanical strength of CF-MPC was calculated according to Equations (1) and (2).

(1) $$R_{c}=\frac{F_{\max}}{A}=\frac{F_{\max}}{a^{2}}$$

where $R_{c}$ represents the compressive strength (MPa), $F_{\max}$ represents the maximum load, A represents the pressure surface area, and a represents the length of the edge of the pressurized surface (40 mm).

(2) $$R_{f}=\frac{1.5F_{\max}L}{b^{3}}$$

where $R_{f}$ represents the flexural strength (MPa), $F_{\max}$ represents the maximum load, L represents the bearing span (100 mm), and b represents the thickness of the sample (40 mm).

## 3. Compression Performance

### 3.1. Failure Modes

**Figure 2:**
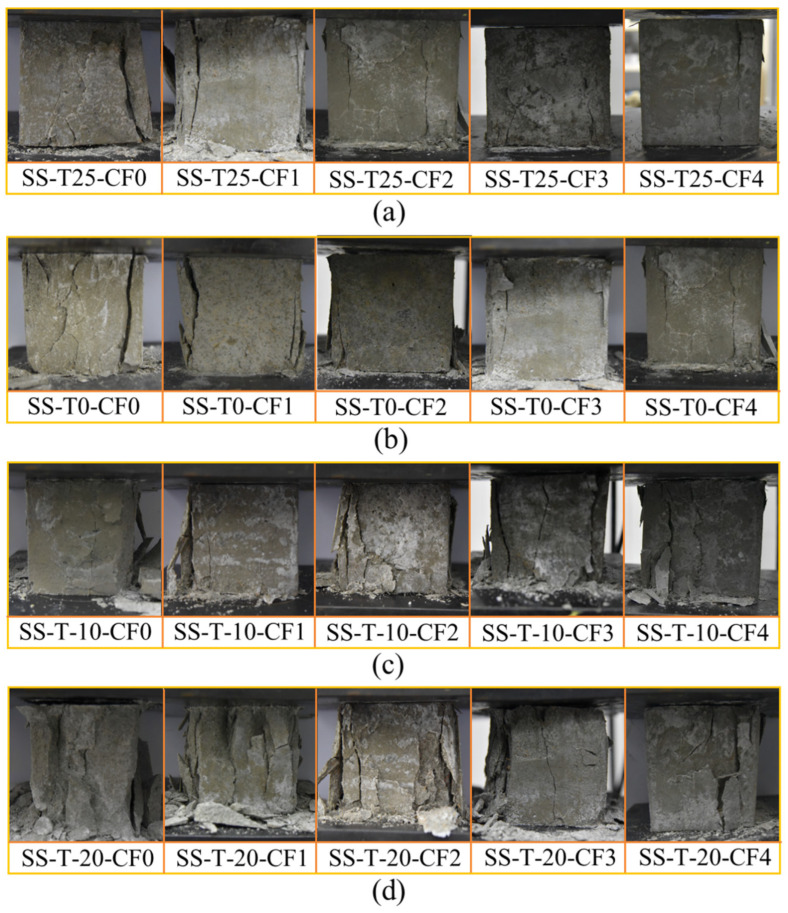
Compression failure modes: (**a**) curing at 25 °C; (**b**) curing at 0 °C; (**c**) curing at −10 °C; (**d**) curing at −20 °C.

Figure 2a shows the failure modes of the specimen when the curing temperature was 25 °C. When the CF content was 0% and 1%, the specimen was loaded to the peak value, the specimen was pressurized until it made a strong crushing sound, and numerous fissures appeared on the surface of the specimen and extended to the interior rapidly until they destroyed the specimen into incomplete fragments. When the CF content was 2%, CF-MPC cracks appeared when the test loaded to destroy the specimen, and a small number of fragments fell off due to the bonding effect of CF at the cracks. When the CF content was 3% and 4%, the plastic deformation capacity of MPC was significantly improved, and although cracks appeared, no specimen fragmentation occurred. Figure 2b shows the compression failure morphology of CF-MPC at 0 °C curing. The trend of CF-MPC damage morphology with the change of CF volume doping was the same as that at 25 °C. As fiber doping increased, there was a decrease in the extent of spalling and fracture extension that occurred when the specimen was damaged. However, the growth of porosity and incomplete hydration within the specimen due to low-temperature curing reduced the compressive capacity of the specimen and caused an increase in crack extension. Figure 2c,d shows the morphology of CF-MPC curing at −10 °C and −20 °C. As the curing temperature declined, the free water in the specimen froze and inhibited the hydration of the MPC. At this time, the strength and deformation resistance of the specimens were severely reduced, causing CF-MPC integrity degradation when damaged. Moreover, under the low-temperature environment, the CF and MPC could not bond well, and adding fibers increased the pore space of the matrix and reduced CF-MPC densification.

### 3.2. Stress–Strain Curve

**Figure 3:**
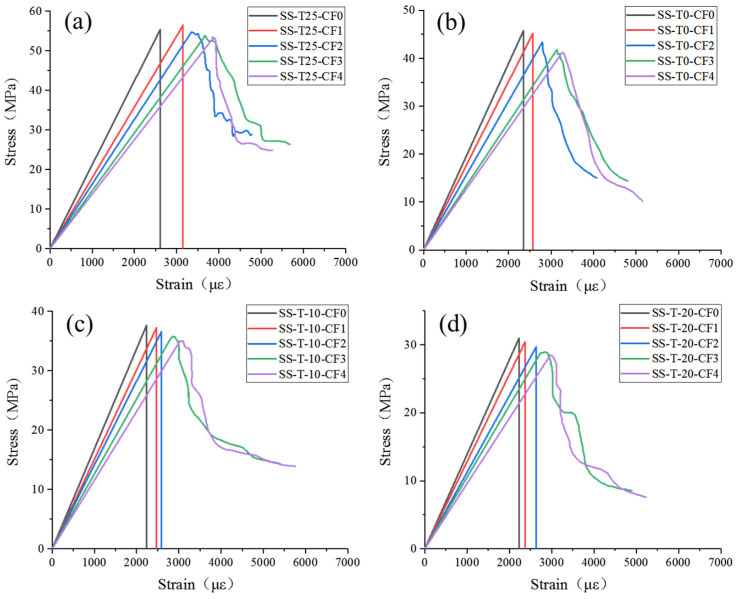
Stress–strain curves of CF-MPC: (**a**) curing at 25 °C; (**b**) curing at 0 °C; (**c**) curing at −10 °C; (**d**) curing at −20 °C.

Figure 3 shows CF-MPC’s stress–strain curves at various maintenance temperatures and CF contents. The curves could be categorized into two phases: approximate linear elasticity and strain softening. In the approximate linear elastic growth stage, the matrix bears the main loading, the bonding between CF and MPC was not apparent, microcracks were generated inside MPC in a stable condition, and longitudinal cracks could be seen on the specimen’s surface when the load hit the peak value. During strain softening, i.e., the descending phase of the curve, the specimen showed soft plastic deformation, and the stress gradually decreased. At this time, the CF and MPC jointly undertook the role of the load, due to their bonding effect, which improved the specimen’s plastic deformation capacity, and delayed the extension of the cracks until the specimen showed a large number of longitudinal cracks and damage under pressure. In addition, when the specimen was pressurized with brittle failure, the stress–strain curve reached the peak value and decreased vertically. As shown in Figure 3a, with the CF content increasing, the peak load of the curvilinear stress fell because a growing number of fibers was replacing the MPC matrix. In addition, it was difficult to distribute the CF uniformly, which led to an increase in the number of pores and microcracks in the specimen [19], a decrease in compactness, and a reduction of the peak stress that can be tolerated. However, at this time, the range of the interval of the strain-softening stage of the specimen became more extensive due to the CF-MPC’s bonding effect, which enhanced its plastic deformation capacity. The compressive stress–strain curves of CF-MPC curing at 0 °C, −10 °C, and −20 °C are shown in Figure 3b–d. Similar to the change in the curve at a curing temperature of 25 °C, the increase in CF content reduced the peak load that CF-MPC could withstand. However, the suppression of specimen hydration levels by low temperatures increased micro cracking of the porosity, weakening the structural integrity of CF-MPC. The structural integrity and strength of the CF-MPC were weakened, and then the peak stress of the specimen under compression decreased with the decline of the curing temperature.

### 3.3. Compressive Strength

**Figure 4:**
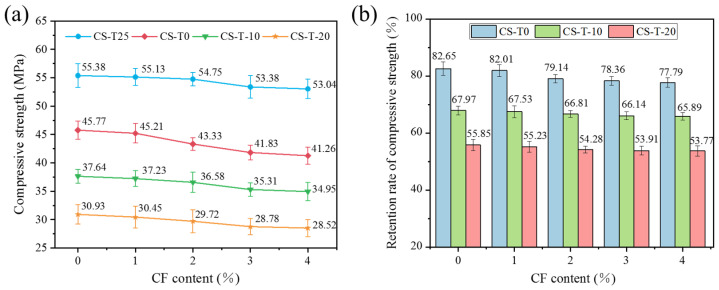
Compressive strength of CF-MPC: (**a**) compressive strength; (**b**) retention rate of compressive strength.

As demonstrated in Figure 4a, both during room temperature and low-temperature curing, the compressive strength of CF-MPC decreased as the fiber content rose, which was because the rise in the amount of the original structure in the MPC replaced by CF led to an overall decrease in the crystal structure of the unhydrated material wrapped by MgKPO_4_-6H_2_O that embodies the strength in CF-MPC. By analyzing Figure 4a,b, it is evident that the specimens’ compressive strength and retention declined to varying degrees as the curing temperature dropped, with the greatest loss occurring between 0 °C and −20 °C. This was because, at low temperatures, MPC moisture crystallizes, forming a hydraulic gradient pressure that expanded inward to form pores and microcracks and the accumulation of hydration products, decreasing CF-MPC compactness and strength. In addition, the strength of the specimens was impacted by the increased porosity and quantity of holes bigger than 10 nm in the CF-MPC due to the low-temperature environment. Additionally, the lower temperature led to incomplete hydration of the specimens, decreasing their compressive strength.

## 4. Bending Performance

### 4.1. Failure Modes

**Figure 5:**
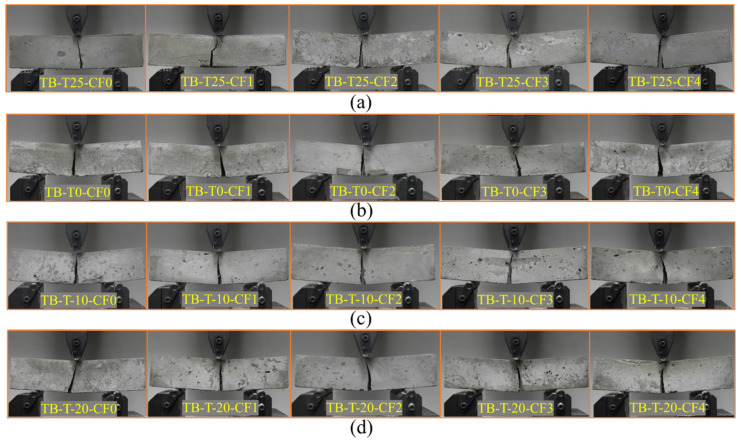
Flexural failure modes: (**a**) curing at 25 °C; (**b**) curing at 0 °C; (**c**) curing at −10 °C; (**d**) curing at −20 °C.

Figure 5a shows the failure modes of the specimen in the bending test when the curing temperature was 25 °C. The results show that the crack width and deflection of the samples at failure grew as the CF content increased. The specimen reached brittle failure when the CF content was 0%. When the test was loaded to the peak value, the longitudinal crack in the middle of the specimen rapidly extended upward until it penetrated through the specimen section. When the CF content was 1%, due to the distribution of fewer fibers in the specimen, the improvement of the flexural performance of the fiber on the matrix was limited, resulting in brittle failure of the specimen as well. The specimen had low toughness failure when the CF content was 2%, and the crack extension rate slowed down during loading. When the test was loaded to the peak value, the specimen was not damaged immediately due to the involvement of CF and could still withstand the load. When the CF content was 3% and 4%, the specimens showed high toughness failure. The cracks developed most slowly during the loading process, and the crack width and bending deflection at the time of specimen damage were higher than those of brittle and low-toughness failures. Figure 5b shows the failure modes of specimens with various CF contents under 0 °C curing. The failure modes of specimens cured at this temperature are similar to those at 25 °C. The brittle failure was observed at 0% and 1% CF content, but the low toughness failure was observed at 2–4%. This was due to the free water crystallization phenomenon of CF after low-temperature treatment, which made the CF surface add holes and deepen cracks, weakening the performance of the fiber. Furthermore, the MPC hydration reaction was slower, and the hydration degree was lower at low temperatures, which reduced the matrix’s strength and the bonding properties of the CF-MPC. Therefore, although the specimens were still able to withstand the load after loading to the peak load, the ability of the specimens to withstand the load was reduced. Figure 5c,d shows the failure modes of specimens with various CF contents under −10 °C and −20 °C curing. The deterioration effect on the performance of the CF, as well as the hydration process and degree of the MPC, was stronger due to the impact of a curing temperature that was too low. The strength and structural compactness of the matrix decreased severely, and the loads the specimens could withstand were subsequently weakened. Brittle failure was observed at CF contents of 0–2%, and low toughness failure was observed at 3–4%.

### 4.2. Load–Displacement Curve

**Figure 6:**
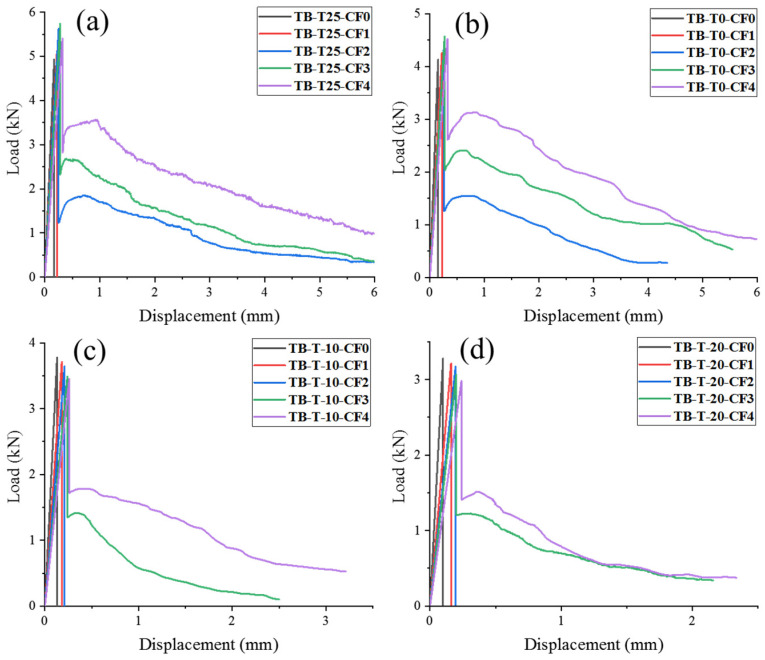
Load–displacement curves of CF-MPC: (**a**) curing at 25 °C; (**b**) curing at 0 °C; (**c**) curing at −10 °C; (**d**) curing at −20 °C.

Figure 6a shows the load–displacement curves for various CF contents under 25 °C curing. When the CF content was 0% and 1%, the curve rose rapidly to peak in the test loading and quickly decreased. This resulted from the high brittleness and poor hardness of the MPC, and it was not evident that 1% CF affected the specimen’s toughness. The specimen quickly fractured in the middle of the span when the peak stress was achieved. When the CF content was 2–4%, the load–displacement curves showed three stages of changes. In the approximate linear elasticity stage, the MPC matrix mainly loaded the specimen, and the curve decreased rapidly after the specimen cracked. At this time, the fibers in the cracks of the specimen were involved in the force, and the crack extension slowed down. Subsequently, the flexural curve entered the stage of stable fracture extension, and the load that could be tolerated rose. As the CF was pulled out and pulled off in the matrix, the curve entered the crack destabilization stage, and began to decrease until the specimen was damaged.

Figure 6b indicates the load–displacement curves of specimens with various CF contents at 0 °C, and the change rule was similar to that at 25 °C. At this curing temperature, the peak load in the near-elastic stage and the stable fracture expansion stage of the load–displacement curves were significantly lower than those of the specimens cured at 25 °C. This was caused by the low-temperature curing’s declining influence on the characteristics and structure of CF-MPC., which made the specimens less resistant to bending. Figure 6c,d shows the load–displacement curves of specimens with various CF contents curing at −10 °C and −20 °C. As the maintenance temperature decreased, the performance of the MPC and CF was more deteriorated than that at 0 °C, and the peak loads in the specimen load–displacement curves in the near-elastic stage and the stable expansion stage of cracks were smaller. At a CF content of 0–2%, the fibers could not enhance the toughness of the specimens due to the weakening of the MPC itself. The specimens showed brittle failure, and the load–displacement curves increased rapidly to the peak load and then decreased abruptly. The toughening effect of CF on the MPC matrix was weaker at CF contents of 3% and 4%. With them, the peak load that the specimen could withstand decreased significantly due to the addition of higher fiber content replacing the matrix’s volume and the fibers’ inability to bond well with the matrix.

### 4.3. Flexural Strength

**Figure 7:**
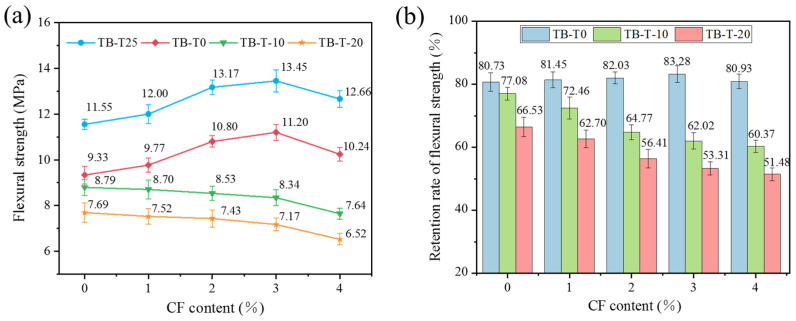
Flexural strength of CF-MPC: (**a**) flexural strength; (**b**) retention rate of flexural strength.

Figure 7 shows MPC’s flexural strength and its retention with various CF dopings at different curing temperatures. According to Figure 7a, while the fiber dosage rose, the flexural strength displayed a rising trend before dropping at 25 °C. The flexural strength of the specimen without fiber doping was 11.55 MPa and reached its maximum value of 13.45 MPa at a 3% CF content, which was credited to the bonding ability between the fiber and matrix, which lessened the MPC’s high brittleness. The decrease in flexural strength when the CF content was 4% was due to the agglomeration of fibers within the MPC, which increased the pores and microcracks in the specimen. When the specimen with CF content was preserved at 0 °C, its flexural strength changed according to a similar changing rule to that at 25 °C, but the bending strength was lower than at 25 °C. This was because, at low temperatures, the MPC hydration rate was slower, and the degree of hydration was incomplete, resulting in a decline in MPC’s strength. Furthermore, as the temperature dropped to less than −10 °C, the flexural strength of the CF-MPC showed a negative connection with a rise in CF content. Specifically, the flexural strength declined from 8.79 MPa to 7.64 MPa under −10 °C curing and from 7.69 MPa to 6.52 MPa under −20 °C curing. The specimens’ preservation of flexural strength at −10 °C and −20 °C curing temperatures was much lower than that at 0 °C, as Figure 7b illustrates for each fiber content. This was due to the rapid free-water freezing in the specimen under negative temperature curing, which caused the MPC hydration rate to be smaller than that under 0 °C curing, and there were more unreacted and under-reacted hydration products in the MPC, which made the internal structure of the specimen looser. Adding CF decreased the MPC’s content and increased the amount of holes and fractures in the matrix, which decreased the strength of the specimen instead of increasing it.

## 5. Microtest Analysis

### 5.1. Hydration Products Analysis

**Figure 8:**
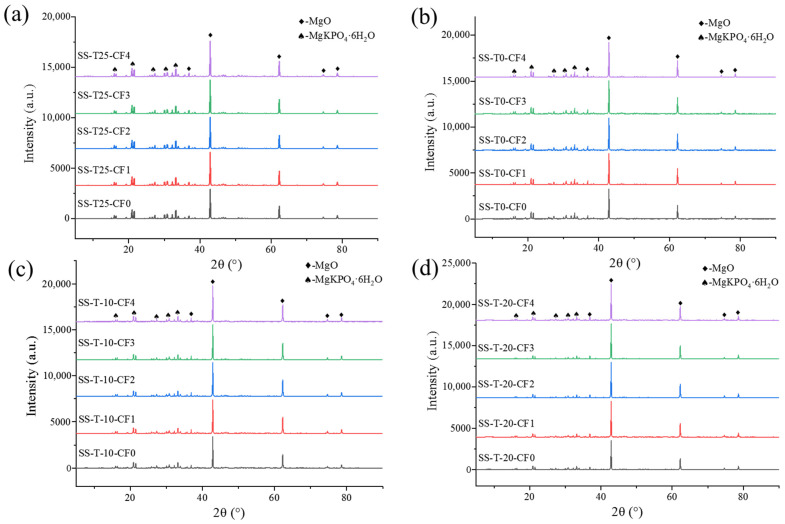
XRD spectra of CF-MPC: (**a**) curing at 25 °C; (**b**) curing at 0 °C; (**c**) curing at −10 °C; (**d**) curing at −20 °C.

The XRD diffraction patterns at various curing temperatures for CF-MPC chemical compositions are shown in Figure 8. It can be found that the diffraction peaks were mainly distributed in the range of 10~80°, and the physical phase was analyzed in the range of the diffraction angle. The primary hydration product of MPC after 28 days at each curing temperature was MgKPO_4_-6H_2_O, and the diffraction angle was more intensive in the range of 15~35°. Furthermore, MgO crystals that were not involved in the hydration event were also present in the matrix, and their main diffraction angles were in the ranges of 40~45°, 60~65°, and 75~80° [32,33].

**Figure 9:**
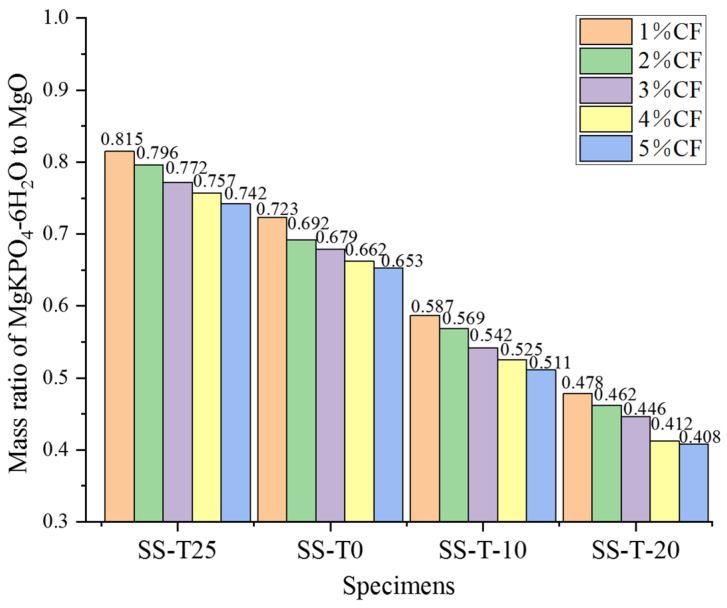
Mass ratio of MgKPO_4_-6H_2_O to MgO under different curing temperatures and CF contents.

The mass ratio of MgKPO_4_-6H_2_O to MgO was obtained by integrating the XRD characteristic peaks as Figure 9 illustrates, which determined the strength of the MPC. As the curing temperature declined, the MgKPO_4_-6H_2_O to MgO mass ratio gradually decreased, which indicates that the amount of unhydrated MgO left behind increased and the production of MgKPO_4_-6H_2_O decreased. This resulted in the MPC hydration process being inhibited by low-temperature curing, which greatly decreased the matrix’s strength. Furthermore, when the temperature dropped, the MPC hydration process was more effectively inhibited. Additionally, because the fibers had some degree of water absorption and too many fibers created the agglomeration phenomena, the hydration reaction was hampered, and the mass ratio of MgKPO_4_-6H_2_O to MgO was a bit lowered with an increase in CF content.

### 5.2. Microstructure

**Figure 10:**
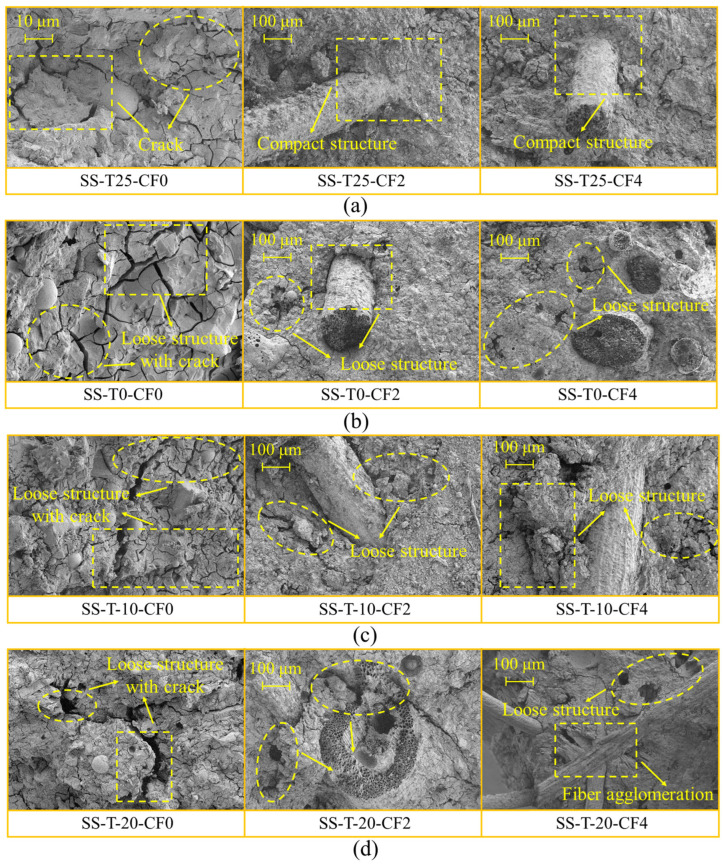
Microstructure of CF-MPC: (**a**) curing at 25 °C; (**b**) curing at 0 °C; (**c**) curing at −10 °C; (**d**) curing at −20 °C.

Figure 10 shows the SEM of the CF-MPC at various curing temperatures, whereas Figure 10a displays the microstructure of the CF-MPC at 25 °C. It can be found that the specimen still produced small cracks, but the overall number of cracks and the crack widths were smaller, and the structure was denser. At this time, the hydration of the CF-MPC was more complete, generating more of the main product, MgKPO_4_-6H_2_O. With the decrease of the curing temperature to 0 °C, 10 °C, and 20 °C, the wider distribution of MPC cracks and the larger width of cracks can be seen in Figure 10b–d, which was because, at low temperatures, the water inside the MPC gradually crystallized and expanded to the internal pores to destroy the internal structure of the MPC. Moreover, with the lowering of the curing temperature, the slower and more incomplete hydration rate of the MPC led to the more rapid inward extension and growth of MPC microcracks, and the structure was looser, which was in line with the findings of the XRD hydration product analysis. In addition, with the increase of CF content, CF appeared in the agglomeration phenomenon so that the pore space of CF-MPC increased, and the structure was less dense, to a certain extent, weakening the CF-MPC.

## 6. Conclusions

In this study, the influence of various fiber contents on the compressive and flexural properties of CF-MPC at different curing temperatures was investigated using static compression test and three-point bending test, respectively. Moreover, XRD and SEM were used to examine the microstructural alterations of CF-MPC. The research findings allow for the following deductions to be made:

(1) Low-temperature curing will alter the CF-MPC’s failure modes in static compression tests, resulting in more cracks and less structural integrity. Lower curing temperatures result in decreased compressive strength for CF-MPC. The compressive strength of CF-MPC without fiber was 30.93 MPa at −20 °C maintenance, which was only 55.85% of that at 25 °C. CF-MPC’s plastic deformation capacity increased as the CF concentration increased, but its compressive strength trended downward.
(2) Low-temperature curing raises the CF content threshold required for the transition from brittle to toughness failure in CF-MPC bending. The flexural strength of CF-MPC exhibited a tendency to rise and then fall with an increase in fiber content, peaking at 13.45 MPa and 11.20 MPa at 3% under curing at 25 °C and 0 °C, respectively. However, when the fiber content rose, CF-MPC’s flexural strength decreased from 8.79 MPa and 7.69 MPa to 7.64 MPa and 6.52 MPa under −10 °C and −20 °C curing conditions.
(3) MPC’s primary hydration product, MgKPO_4_-6H_2_O, declined with a decreasing curing temperature, whereas the remaining quantity of hydration reactant MgO rose, according to XRD spectra of CF-MPC’s hydration products at different curing temperatures. These phenomena suggest that the main cause of the decline in CF-MPC’s strength is a reduction in the hydration degree of MPC with decreasing temperature.
(4) As the curing temperature decreased, the pores and microcracks of the CF-MPC increased, and the specimen’s structure became looser, at which time the bonding ability of CF and MPC was weaker. In addition, the addition of too many fibers also led to the increase of pores and microcracks in the specimen.

## Figures and Tables

**Table 1:** Chemical composition of MgO and FA (%).

Component	MgO	SiO_2_	CaO	SiO_2_	K_2_O	Fe_2_O_3_	Al_2_O_3_	Loss
MgO	96.25	—	1.18	1.16	—	1.09	0.29	0.03
FA	—	54.94	2.63	54.94	1.76	2.52	34.86	3.29

**Table 2:** MPC mix ratio parameters.

MPC Components				Water-to-Cement Ratio (%)
MgO	KH_2_PO_4_	FA	Na_2_B_4_O_7_-10H_2_O	
1.0	0.68	0.25	0.1	15

## Data Availability

Data are contained within the article.
